# Mapping a candidate gene (MdMYB10) for red flesh and foliage colour in apple

**DOI:** 10.1186/1471-2164-8-212

**Published:** 2007-07-03

**Authors:** David Chagné, Charmaine M Carlisle, Céline Blond, Richard K Volz, Claire J Whitworth, Nnadozie C Oraguzie, Ross N Crowhurst, Andrew C Allan, Richard V Espley, Roger P Hellens, Susan E Gardiner

**Affiliations:** 1 The Horticulture and Food Research Institute of New Zealand (HortResearch) Palmerston North, PB 11030, Manawatu Mail Centre, Palmerston North 4442, New Zealand; 2 HortResearch Hawke's Bay, PB 1401, Havelock North 4157, New Zealand; 3 HortResearch Mount Albert, PB 92169, Auckland 1142, New Zealand

## Abstract

**Background:**

Integrating plant genomics and classical breeding is a challenge for both plant breeders and molecular biologists. Marker-assisted selection (MAS) is a tool that can be used to accelerate the development of novel apple varieties such as cultivars that have fruit with anthocyanin through to the core. In addition, determining the inheritance of novel alleles, such as the one responsible for red flesh, adds to our understanding of allelic variation. Our goal was to map candidate anthocyanin biosynthetic and regulatory genes in a population segregating for the red flesh phenotypes.

**Results:**

We have identified the *Rni *locus, a major genetic determinant of the red foliage and red colour in the core of apple fruit. In a population segregating for the red flesh and foliage phenotype we have determined the inheritance of the *Rni *locus and DNA polymorphisms of candidate anthocyanin biosynthetic and regulatory genes. Simple Sequence Repeats (SSRs) and Single Nucleotide Polymorphisms (SNPs) in the candidate genes were also located on an apple genetic map. We have shown that the MdMYB10 gene co-segregates with the *Rni *locus and is on Linkage Group (LG) 09 of the apple genome.

**Conclusion:**

We have performed candidate gene mapping in a fruit tree crop and have provided genetic evidence that red colouration in the fruit core as well as red foliage are both controlled by a single locus named *Rni*. We have shown that the transcription factor MdMYB10 may be the gene underlying *Rni *as there were no recombinants between the marker for this gene and the red phenotype in a population of 516 individuals. Associating markers derived from candidate genes with a desirable phenotypic trait has demonstrated the application of genomic tools in a breeding programme of a horticultural crop species.

## Background

The molecular basis of red, anthocyanin-derived, colour is one of the most studied traits in model plant species [1], partly because of the abundance and ease of visual screens for phenotypic mutations. Moreover, the structural genes involved in the anthocyanin biosynthesis pathway, and the genes regulating this pathway, have been well described [2-6]. DNA polymorphisms located in sequences of both biosynthetic and regulatory genes have been reported to co-segregate with red colour phenotype in several plant species. For instance, within the *Solanaceae *family, candidate genes underlying red colour phenotypes were identified, through sequence similarity, to functionally characterised genes from tomato and petunia [7]. The *A *locus that controls anthocyanin accumulation in the foliage, flower and young fruits of pepper was found to co-segregate with a R2R3 MYB transcription factor (TF). The potato *P*, *R *and *I *loci were identified as being a flavonoid 3',5'-hydroxylase, a dihydroflavonol 4-reductase and a MYB TF, respectively [7]. Similar findings were also reported in cereals, where another MYB TF was shown to control red grain colour [8]. More recently, the allelic diversity within a segregating population of *Arabidopsis thaliana *showed linkage between an AtMYB75 allele and the sucrose-induced accumulation of anthocyanin [9]. In *Antirrhinum *[10], petunia and maize [7,11,12], allelic diversity of certain MYB genes are key determinants in the inheritance and segregation of altered anthocyanin distribution.

Altered expression of several genes with sequence similarity to anthocyanin biosynthetic genes has been associated with red skin colour in apple (*Malus *× *domestica *Borkh.) [13,14], and a gene with sequence similarity to the MYB-class of transcription factors, known to regulate the biosynthetic steps in anthocyanin metabolism, has been described and functionally characterised [15,16]. An R2R3 MYB TF named MdMYB10 showed high levels of expression in red-fleshed fruit and induced red colour when transiently infiltrated into tobacco leaves. Red foliage was also observed when a cDNA of the MdMYB10 was over-expressed in transgenic 'Royal Gala' apple. Recently, a gene with over 99% sequence identity to MdMYB10 (MdMYB1) was shown to co-segregate with fruit skin colour in a population of 136 plants [15], demonstrating that allelic variation of MdMYB10-like genes are associated with alleles that influence anthocyanin distribution.

The availability of an Expressed Sequence Tag (EST) dataset for apple [17] made it possible in this study to identify candidates with high sequence similarity to functionally characterised genes (both structural and regulatory) found in model species. Microsatellites and sequence variations corresponding to single nucleotide polymorphisms (SNPs) are often encountered in contigs assembled from EST datasets, as have been shown in several crop species where large EST datasets are available [18]. Mapping molecular markers located within, or proximal to, candidate genes for which both the function and expression are known is very attractive, and can lead to the identification of the gene controlling the trait of interest.

While there are reports on the chemical components of red colour in apple [19], few studies assessing the genetic control of this trait have been published. Lewis and Crane [20] concluded that red foliage colour was controlled by a single major dominant gene, Trebuschenko [21] suggested that foliage colour was controlled by two genes. Similarly, the genetic control of fruit skin colour has been debated. Red colouration in the skin was suggested to be controlled by either two dominant genes *A *and *B*, with *A *linked in coupling phase with a third gene, *L*, controlling the degree of blush [22], or by a single locus *Rf *[23]. A molecular marker linked to the *Rf *locus controlling skin colour has been developed [23] and was mapped to LG 09 [24]. Mendelian loci controlling colour have been characterized in other Rosaceous tree crops in the *Prunus *genus [25]. Natural red leaf mutants of pear are also thought to be regulated by a single dominant gene [26,27].

Apple cultivars present a wide range of colour variation in the fruit skin, from green such as 'Granny Smith', through to partially coloured apples, such as 'Royal Gala' (striped) or 'Sciros' (Pacific Rose™) (blushed), through to dark red such as 'Red Delicious'. Red colour varies not only in the fruit skin, but also in other plant parts such as flowers, foliage, woody organs and fruit flesh. Red flesh colour is a highly desirable trait: consumer studies have shown that there may be a premium for fruit with novel colour distribution [28]. In addition to a high fresh fruit market value, red pigments, which are mainly anthocyanins [29], represent an excellent source of antioxidants, with potential health benefits [30-33]. Although all the most popular commercial apple varieties have white flesh, some named varieties, such as 'Pink Pearl' and 'Weirouge' show varying levels of red pigmentation in their flesh [19]. Pigmentation in red flesh may be associated with poor quality of high astringency, small fruit size and soft texture, such as found in crab apples (*Malus *spp.). One of the objectives of our apple breeding programme is to introduce the red flesh trait into high quality commercial cultivars. The development of cultivars with commercial potential can be accelerated using marker-assisted selection (MAS). For this purpose, it is very useful to understand the genetic basis and segregation of red pigmentation in apple flesh.

Our goal was to identify markers in candidate genes and test them for co-segregation with the red flesh phenotype, using segregating populations. For most of the candidate genes present in our database, we were able to identify variable DNA sequence features that enabled us to locate them on the apple genetic map. We were able to support the data that MYB10-like genes play key roles in the molecular determination of anthocyanin distribution and demonstrate that the MYB10 gene is associated with an allele responsible for red flesh and foliage in apple.

## Results

### Phenotypic segregation for red colour

Two populations were assessed for colour in the fruit core and cortex. In the A194 family ('Sciros' × 91.136 B6-77), of the 163 fruiting individuals, 31 plants bore fruit with red core and cortex. A proportion of the population (38 plants) exhibited a red cortex/white core phenotype, and the remaining 94 plants were completely white-fleshed. These results were consistent in each of the three years of the study. In a second population, 'Geneva' × 'Braeburn', 31 of the progeny had fruits with red core and cortex, while the remaining 30 progeny that were phenotyped had white fruits, corresponding to a 1:1 Mendelian ratio (χ^2 ^= 0.02).

The foliage colour was assessed for 32 segregating apple seedling families (8,759 plants in total) resulting from controlled pollination (Table 1). The foliage colour segregation ratio for the pooled data was 2,940:5,819 (red:green), which is close to 1:2 (χ^2 ^= 0.285). However, segregation ratios showed no clear trend across the 32 families analysed, nor across families with a common red-leafed father. Approximation of the segregation ratios ranged from 1:1 for 4 families to 1:3 for another 11 families, and in one case ('Scired' × 94.136 K5-119) as high as 1:13, always favouring green leaf.

**Table 1:** Description of the 32 families used for studying the genetic determinism of red foliage colour.

**Year crossed**	**Mother**	**Father**	**Number of red leafed progeny**	**Number of green leafed progeny**	**Total number of progeny**	**χ^2 ^test **			
						**1:1**		**1:3**	
1998†	'Sciros'	91.136 B6-77	121	395	516	145.5	***	1.0	

1999	NZ-selection1	91.136 B6-77	620	724	1344	8.0	***	480.1	***

2000	NZ-selection2	91.136 B6-77	137	390	527	121.5	***	0.4	

	'Royal Gala'	91.136 B6-77	24	26	50	0.1		21.2	***
	'Sciros'	91.136 B7-66	10	53	63	29.3	***	4.2	*
	'Scired'	91.136 B7-66	7	79	86	60.3	***	19.6	***
	'SciGold'	91.136 B7-66	77	207	284	59.5	***	1.0	
	'Granny Smith'	94.036 K3-84	15	26	41	3.0		4.4	*
	'Royal Gala'	94.036 K3-84	16	31	47	4.8	*	3.1	
2001									
	'Sciros'	94.036 K5-119	3	33	36	25.0	***	8.0	***
	'Scired'	94.036 K5-119	13	171	184	135.7	***	47.3	***
	'SciGold'	94.036 K5-119	34	170	204	90.7	***	11.3	*
	'Granny Smith'	92.087 D36-51	19	35	54	4.7	*	4.5	*
	'Royal Gala'	92.087 D36-51	88	135	223	9.9	***	37.3	***
	'Royal Gala'	93.014 J12-09	73	265	338	109.1	***	3.1	

	NZ-selection3	NZ-selection8	111	285	396	76.5	***	2.9	
	'SciGold'	NZ-selection8	129	210	339	19.4	***	46.2	*
2003	NZ-selection4	NZ-selection8	28	93	121	34.9	***	0.3	
	NZ-selection3	NZ-selection9	174	382	556	77.8	***	17.6	*
	'SciGold'	NZ-selection9	432	493	925	4.0	*	348.5	*

	NZ-selection5	91.136 B6-77	34	53	87	4.1	*	13.8	***
	NZ-selection6	91.136 B6-77	22	69	91	24.3	***	0.0	
	NZ-selection7	91.136 B6-77	40	108	148	31.2	***	0.5	
	NZ-selection3	91.136 B6-77	12	49	61	22.4	***	1.4	
	'Fuji'	91.136 B6-77	20	93	113	47.2	***	4.8	*
	'Scired'	91.136 B6-77	56	334	390	198.2	***	35.3	***
2004									
	'Sciros'	91.136 B6-77	40	124	164	43.0	***	0.0	
	NZ-selection4	NZ-selection10	129	227	356	27.0	***	36.0	***
	'Fuji'	NZ-selection10	30	53	83	6.4	*	8.2	***
	'Sciros'	NZ-selection10	336	380	716	2.7		275.4	***
	'Elstar '	'Geneva'	45	80	125	9.8	***	12.1	***
	'Geneva'	'Braeburn'	45	46	91	0.01		43.5	***

The red-leafed 91.136 B6-77, NZ-selection8, NZ-selection9, NZ-selection10 and 'Geneva' all have *Malus pumila *var. *niedzwetzkyana *in their pedigrees. 'Geneva' is an open-pollinated seedling of *Malus pumila *var. *niedzwetzkyana*, NZ-selection8 and NZ-selection9 are seedlings from a 'Royal Gala' × 'Geneva' cross. NZ-selection10 is a seedling of a ('Royal Gala' × 'Prima') × 91.136 B6-77 cross. The origin of red foliage colour in 94.036 K-84, 92.087 D35-51 and 93.014 J12-09 is unknown. The green-foliaged breeding parents NZ-selection2, NZ-selection3, NZ-selection4, NZ-selection6, NZ-selection7, and 'SciGold' are from a 'Royal Gala' × 'Braeburn' cross, NZ-selection5 from a 'Red Delicious' × ('Gala' × 'Splendour') cross and NZ-selection1 from a 'Royal Gala' × 'Prima' cross. †: A194 population used for genetic map construction. χ^2 ^test for segregation: * P(χ^2 ^> 3.84) = 0.05; *** P(χ^2 ^> 7.87) = 0.005.

In both the A194 and the 'Geneva' × 'Braeburn' families, all the trees that bore red cored fruits had red leaves and a red cortex, while the plants that bore completely white-fleshed fruits were green-leafed. In the A194 population, the individuals that exhibited a white core/red cortex phenotype were green-leafed. All the red foliage parents used in the 32 populations bore red core fruit.

### SSR and SNP discovery

A total of 268,168 apple EST sequences, including those described by Newcomb *et al*. [17], and those deposited in the GenBank database, were assembled into contigs and preliminary annotation performed by sequence homology (blastx v. *Arabidopsis *proteins [34]). Twelve out of the 13 candidate genes for colour [16] were represented in the database (Table 2). Full cDNA sequence of the remaining candidate, MdMYB10, is described elsewhere [16]. For seven of the 13 candidate TF and biosynthetic genes, contigs were assembled from sequences obtained from more than eight cDNA clones at a clustering threshold of 95%.

**Table 2:** SNPs and SSRs detection in the candidate genes using *in silico *detection (a) and re-sequencing (b).

**a)**								
					
	**Number of cDNA clones in the contig alignment (number of cultivars)**	**Contig size (bp)**	**Number of SNPs**					
					
MdBHLH3	8 (3)	2,717	19					
MdTTG1	10 (5)	1,575	2					
MdBAN	51 (12)	1,468	21					
MdCHS	87 (9)	1,548	29					
MdDFR	21 (7)	1,542	6					
MdF3H	30 (6)	1,388	3					
MdANS	19 (5)	1,468	23					
					
Total	-	11,706	103					
					
**b)**								
			**Intron**		**Exon**			
						
**Gene ID**	**Number of cDNA clones in the contig alignment (number of cultivars)**	**Size of the PCR fragment sequenced (bp)**	**Size (bp)**	**Number of SNPs**	**Size (bp)**	**Number of SNPs**	**Total number of SNPs**	**SSR**

MdMYB10	na	1,945	1,331	9	614	2	11	-
MdMYB9	2 (2)	606	203	0	403	1	1	CT_15_
MdMYB17	3 (2)	1,392	1,010	0	382	0	0	T_14 _-- AT_10_
MdBHLH33	4 (3)	1,168	593	6	575	4	10	-
MdANL2	4 (3)	635	137	0	498	0	0	-
MdMYB12	5 (2)	730	223	4	507	4	8	TGTA_14_
MdBHLH3	8 (3)	522	99	0	423	0	0	CT_15_

Total	-	6,998	3,596	19	3,402	11	30	4

A SNP detection tool identified a total of 103 SNPs within the seven genes with more than eight cDNAs, over a cumulative sequence length of 11,706 bp (Table 2a). This corresponds to a frequency of one SNP every 113 bp. In the remaining five candidate genes, for which a contig was identified in the database, the number of sequences in each alignment ranged from two to five ESTs only (Table 2b) and was not sufficient to ensure authentic SNP detection. For these, as well as for MdBHLH3 and MdMYB10, cloning and sequencing of PCR fragments obtained from amplification of genomic DNA from the parents 'Sciros' and 91.136 B6-77 resulted in detection of a further 30 SNPs in four genes of the set of five (Table 2b), from 3,596 bp of intron and 3,402 bp of exon sequences respectively, corresponding to a frequency of one SNP every 233 bp. No SNPs could be detected in MdANL2. Two insertion-deletions (indels) of four and 51 bp were detected for MdMYB10 intron and MdBHLH3 exon, respectively.

Dinucleotide SSRs were detected in four out of 13 candidate genes. One was located in the 5' untranslated region (UTR) of MdBHLH3 and three were in the introns of MdMYB9, MdMYB17 and MdMYB12.

### Marker development and genetic mapping

**Figure 1:**
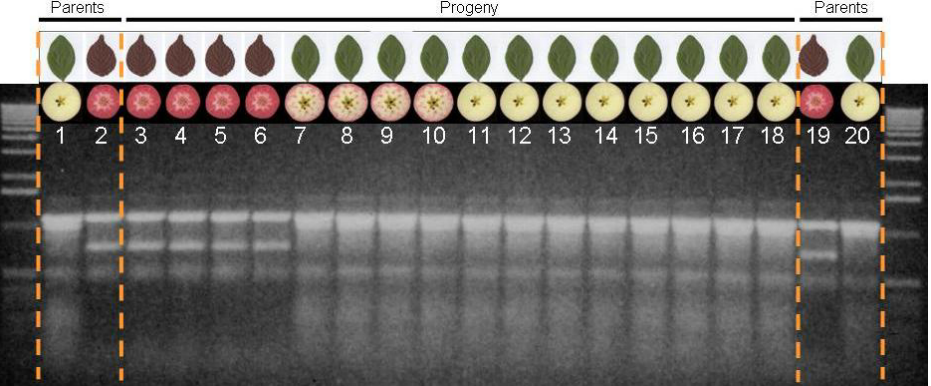
**Co-segregation between the MdMYB10 marker and red core and foliage**. The marker developed for MdMYB10 (indel) was screened over a subset of the A194 population (16 individuals) and both parents. Lanes 1 and 20, and lanes 2 and 19 correspond to the parents 'Sciros' and 91.136 B6-77, respectively. Three phenotypes were observed for fruit flesh colouration in the progeny: a/red cortex and core (lanes 3 to 6); b/white core and red cortex (lanes 7 to 10); c/white cortex and core (lanes 11 to 18). All the fruit with red core were borne on red foliage trees and showed a 786 bp band for the MdMYB10 marker, the two other types are green-leafed and lacked the 786 bp band. A 1,182 bp PCR product was used as a positive control for PCR amplification. 1 Kb Plus DNA ladder (Invitrogen, Carlsbad, CA, USA) was run alongside the PCR products.

Although three of the four SSR markers were polymorphic in the population segregating for red flesh, none of them co-segregated with red colour. Five out of seven SNP markers were polymorphic between the parents of the population. The SNP marker developed for MdCHS gave a two-band monomorphic profile, suggesting the presence of another copy of the gene and that the SNP was not allelic but because of a polymorphism between these two loci, this is perhaps not surprising given that the chalcone synthase has also been described as a gene family in species such as petunia [35] and pea [3]. The SNP marker developed from MdBHLH33 EST sequence was monomorphic with a single amplification product, suggesting that this SNP was not present in either of the parents of the cross analysed. One marker, developed from the four base pairs indel present in the MdMYB10 intron, produced a 786 bp PCR product which segregated in the A194 population and was only present in red fruit core/red-leafed individuals (Figure 1). None of the other markers exhibited co-segregation with red flesh (core or cortex). The marker developed from the MdMYB10 indel segregated in a 3:1 ratio (χ^2 ^= 1.0), although the parental genotypes (i.e. present for 91.136 B6-77 and absent for 'Sciros') and sequencing data (i.e. ATAA/- × ATAA/ATAA) predicted a 1:1 ratio.

**Figure 2:**
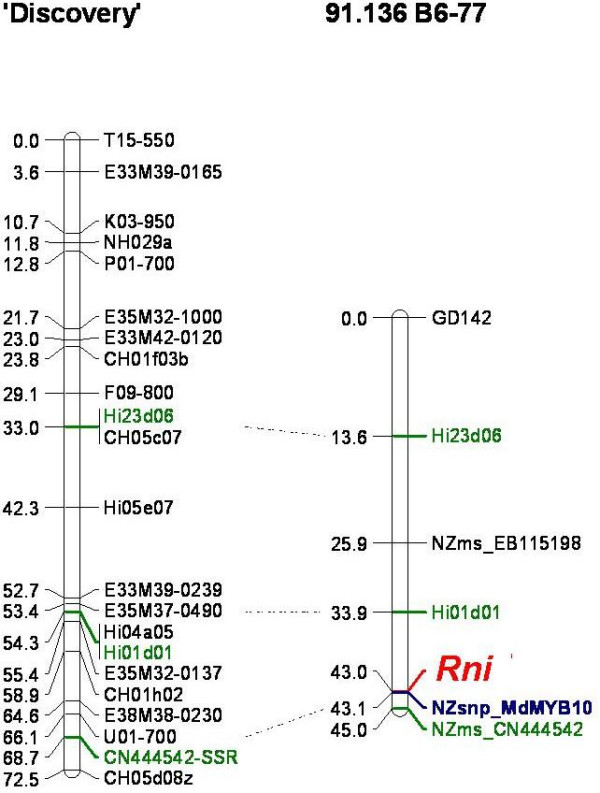
**Genetic map of apple linkage group 09 showing the position of *Rni *and MdMYB10**. The genetic maps of 'Discovery' (left) [36] and 91.136 B6-77 (right) aligned with three common microsatellite markers (green). *Rni *(red) and MdMYB10 (blue) are located at the bottom of the linkage group. NZms_EB115198 is unpublished (E. Rikkerink, S. Gardiner, K. Zuiderdin, D. Chagné, J-M. Celton).

After screening the markers across the progeny of a 'M.9' × 'Robusta 5' cross for which a genetic map is being constructed (J-M. Celton and S.E. Gardiner, personal communication), we assigned a linkage group to five of the candidate genes (Table 3). MdDFR remained unlinked (LOD score < 3) and the remaining markers were monomorphic in this population. The MdMYB10 indel marker did not segregate in this progeny set, nor in three other mapping populations available to us (data not shown). For that reason, SSRs markers from reference maps with defined linkage group locations were screened over the A194 population. Because a red skin marker was located on LG 09 [24], markers from this LG were screened first. Four published SSRs, CN444524-SSR, Hi23d06, Hi01d01 [36] and GD142 [37], and the MdMYB10 indel were screened over the whole A194 population and a genetic map of LG 09 was constructed for the parent 91.136 B6-77 (Figure 2). Linkage was detected between MdMYB10 and the LG 09 markers. MdMYB10 was located between Hi01d01 (9.1 cM, LOD 29.1) and NZms_CN444542 (2 cM, LOD 49.3). The use of transferable SSR markers made it possible to then align the genetic map developed in the A194 population with the published map of 'Discovery' [36] and to assign MdMYB10 to the bottom of LG 09.

**Table 3:** List of candidate genes studied and marker information

**Gene type**	**Gene ID**	**Marker type**	**Forward primer**	**Reverse primer**	**PCR product (bp)**	**LG**
	MdMYB10	Indel	CGAGTGCTAATAGCAACC	CCTCTGTTGGCCGAATACAC	786	9
			AATGGCCTACCACATCATTG		1,182	
	MdMYB9	SSR	TCCATAACCTTCTTGGTAACAG	AAGGAGAATCGGCTTTCGAT	329	NP
	MdMYB17	SSR	TGCTCCTCTCTAGCTATTGCATAAT	AAGACTCACAAACTAGCTGTCAAAT	205	14
Transcription factors	MdBHLH33	SNP	TGGCATTCAGCTAAAACC*T *AAT	TCTCTGGCACCCTCTCTTGG	145	NP
	MdANL2	-	-	-	-	-
	MdBHLH3	SSR	CAACTCCCCTTATTCTTCTTCTCTC	CACCTGACCTTCTCTCTACCTCTAC	170	11
	MdMYB12	SSR	CTCGGCAATCGGTAAAGCTA	TATGAACAGTGAAACCCTAACCCTA	152	3
	MdTTG1	SNP	CACCCGAACCCCAACG*G *CCA	CTCTCCAGCTCCGCCACTG	527	15
	
	MdBAN	SNP	GTTGGGTGAGCTAGAGATATTC	GATACTGCTGGGAACGTC*A *GGA	472	10
	MdCHS	SNP	GAGGAAGTTCGCAAGGCTCA	CCCCATTCCTTGATGGCT*A *TGA	343	NP
Flavonoid pathway	MdDFR	SNP	CTGTGAATGTGGAGGA*G *GAC	TCAGTTTTCTCTGCCGGAAT	616	UL
	MdF3H	SNP	GGCGTGAAATTGTGACCTACT*A *TTT	TGACCACAAAAGCTCCTTCC	392	NP
	MdANS	SNP	TCAGACCTAAAGATGA*C *CTT	TCCTGACCTTGTCCATGAGC	1,449	NP

NP: not polymorphic in the M.9 × 'Robusta 5' population. UL: unlinked. LG: linkage group. The SNP position in the allele-specific primer is underlined. Extra mismatch in the allele-specific primer is shown in italics. No marker could be designed for MdANL2, because neither SNPs nor SSRs were detected.

## Discussion

### Genetic determination of red flesh and foliage

Out of 163 and 61 fruiting trees in the 'Sciros' × 91.136 B6-77 (A194) and 'Geneva' × 'Braeburn' populations, respectively, all the red core fruit were harvested from red foliage trees, indicating that those two characters may be controlled by the same (or a closely linked) gene. In the A194 population, the segregation ratio of 1:3 (Table 1) suggests that at least two dominant genes control both red foliage and red core. This appeared to be in agreement with the hypothesis of a two-gene model suggested by Trebuschenko [21]. However, the absence of any recombinants between the marker for MdMYB10 and red foliage phenotype among 516 individuals suggests that there is actually only one gene controlling this character in the A194 family, consistent with the model purposed by Lewis and Crane [20]. The apparent segregation ratio of 1:3 in the A194 family, as well as the variable segregation patterns of foliar colour observed in all other 31 families (Table 1) was also noted in some of Trebuschenko's red-foliaged families [21]. This suggests that a distorted segregation prevalent in populations derived from red-foliaged parents may be responsible for the difference observed between phenotype and that predicted inheritance pattern from a dominant MdMYB10 allele. This is confirmed by the fact that the closest SSR marker (NZms_CN444542) also showed a segregation distortion. However, it would be interesting to genotype the germplasm used by Trebuschenko to make sure that there is not a second allele that can describe the inheritance observed.

Segregation distortions have been noted previously in other apple mapping populations [24,38]. Those involving the *Vf *resistance gene in apple were inferred to be caused by the presence of several sub-lethal genes, some of which were closely linked to *Vf*. [39]. The variable segregations in foliage and flesh colour observed amongst families in our study (Table 1) was also found in families segregating for *Vf *[34]. The reason for this variability is not understood but may occur because several sub-lethal genes are required in partnership for lethality to occur. As these can be supplied both by donor (linked to the major gene) and by the recurrent parent, their segregation in a family will depend upon their allelotype in each of the parents.

The lethality factor in *Vf *families seems to be caused by dwarfing and poor vigour and eventual plant death. However, germination and survival rates of plants up to five weeks old were high (> 88%) in our red foliage families assessed in 2004 and similar to other green × green-leafed families growing at the same time (data not shown). Similarly, an earlier study concluded that germination rates were the same for green and red pigmented apple seed [20]. It is therefore unlikely that the distortions in inheritance of the foliage colour that occurred in our families resulted from a variable survival rates of the seeds planted. This suggests that the distortion linked with red foliage may be due to an effect at fertilisation or during seed formation.

As 'Redfield', the grandparent of the population segregating for the presence of the red core and red foliage phenotype, is derived from the accession *Malus pumila *var. *niedzwetzkyana*, we named this locus *Rni *(i.e. *R *for red and *ni *for *niedzwetzkyana*). We identify a second phenotype (i.e. green foliage/red fruit cortex/white fruit core) carried by 91.136 B6-77, and propose that a second, independent allele controls the red cortex/white core, but further studies are required to determine its exact nature.

### Candidate gene mapping in apple

Most of the previous mapping studies involving simply inherited or quantitative traits in apple have been carried out using anonymous markers, either bulked segregant analysis [40] with RAPD markers, whole genome scans using microsatellites [41], or quantitative trait locus (QTL) analysis on saturated genetic maps [42]. Our present study followed an alternative route that used the genomics tools newly developed for apple [17]. Our approach is based on the mapping of candidate genes with sequence similarity to genes of known functions. This genomics approach has made it possible to build on the functional genomics that has taken place in model plants and provides a bridge between crop genomics and fruit breeding technologies.

We have identified a polymorphism located in MdMYB10 that co-segregates with the presence of red core colour in apple flesh and red foliage. This result, in combination with previous observations using expression studies and transformation in model systems [16], strongly indicates that MdMYB10 is one gene controlling red colour in apple. We have also previously shown that up-regulation of the MdMYB10 gene is responsible for the elevated accumulation of anthocyanin in red-fleshed apples [16]. Expression of MdMYB10 regulates the expression of the biosynthetic genes in the anthocyanin pathway; CHS: chalcone synthase, CHI: chalcone isomerase, F3H: Flavonol 3 hydroxylase, DFR: dihydro flavonol reductase, LDOX: leucoanthocyanidin dioxygenase and UFGT: flavonoid 3-O-glucosyl transferase. Indeed, these genes have been shown to follow the same expression pattern as MdMYB10 in red v. white flesh apple fruits [16]. SNP markers developed for DFR and CHS did not show any co-segregation with red colour indicating that while these genes may be necessary for anthocyanin biosynthesis, allelic variants are not responsible for the red-flesh phenotype under investigation. This phenotype could, however, be achieved either through an alteration in the regulation of the MdMYB10 gene or by an alteration in the expression of a closely linked gene that regulates MdMYB10. Demonstrating the co-segregation of MdMYB10 with the inheritance of the red fruit core phenotype suggests that the altered expression of the MdMYB10 is the result of a change either in the upstream regulatory region of MdMYB10, elevating its expression in apple fruit, or in its RNA secondary structure, altering the RNA stability of the MdMYB10 transcript of the red-fleshed allele. Although simply inherited traits are often due to a mutation in structural genes, mutations located in regulatory genes, such as TFs, can also account for qualitative variations in agronomic traits [7-9]. Another interesting observation is the fact that the red foliage/red fruit core trees also produce red flowers, bark and wood, indicating that alteration in transcription or post-transcription of MdMYB10 has resulted in a regulatory effect that targets elevated levels of RNA accumulation in the whole plant or in a constitutive manner.

BC226, a marker linked to *Rf *(i.e. less than 2 cM)[23] which controls skin colour in apple, was mapped to LG 09 [24]. Unfortunately, BC226 was monomorphic in our A194 population and so could not be positioned on the same map as MdMYB10. However, the fact that they are both located at the bottom of LG 09 suggests that *Rf *and *Rni *could be located in a gene cluster or even correspond to alleles of the same gene. A MYB TF homologous to MdMYB10 and named MdMYB1 was recently characterized and was shown to co-segregate with skin colour in a population of 136 individuals from a cross between a sibling of 'Cripp's Pink' and 'Golden Delicious' [15]. Further work is needed in order to ascertain whether MdMYB10 controls skin colour in apple, as well as foliage and fruit core. Interestingly, fruit skin colour (*Sc*) and leaf colour (*Gr*) collocate in *Prunus *[25], which suggests there may be a region of synteny between the middle of *Prunus *LG 06 and the bottom of *Malus *LG 09. The isolation and mapping of orthologues of MdMYB10 in other Rosaceae, including *Prunus *species, could help to validate the control of red colour and confirm the finding of a syntenic region.

Although we did not identify any recombinants between *Rni *and MdMYB10 in the A194 population, further work is needed to validate their complete co-segregation. Preliminary validation results show that the MdMYB10 markers co-segregates with foliage colour in other mapping populations; however, family genetic mapping studies, even when using large segregating populations, cannot rule out linkage at a very close physical distance, because of the low probability of detecting a recombination at a small distance (in apple 1 cM is equivalent to 600 kb on average). Therefore, this research has not completely ruled out that the gene underlying *Rni *could be another gene physically closely linked to MdMYB10, although our previous functional results [16] make this less likely. A more effective way to test and validate the genetic control by MdMYB10 of red colour might be to use association studies [43,44].

## Conclusion

We provide genetic proof that the presence of red flesh (fruit core) and red foliage in apple can be determined by a unique single locus and we have named one allele controlling the red colour *Rni*, on the basis of its origin from *M. pumila *var. *niedzwetzkyana*. A molecular marker derived from MdMYB10, which is able to control red colour in apple, co-segregates with the *Rni *locus that we have positioned on LG 09 of the apple genome, a region previously implicated in controlling red skin colour. A second gene controlling red cortex in the absence of red foliage remains to be identified, and based on the success of the present studies, we believe that the candidate gene approach would also be an appropriate route for the discovery the underlying molecular basis of this allele.

## Methods

### Plant material

A total of 32 families were used to dissect the genetic basis of red flesh and foliage colour in apple (Table 1). The crosses were made between 1998 and 2004 and resulted from hybridisations between commercial cultivars (mother tree) and red flesh pollinators, except for a 'Geneva' × 'Braeburn' cross made in 2004 where the mother tree possessed the red phenotype. The F_1 _population A194 ('Sciros' × 91.136 B6-77) consisting of 516 individuals and segregating for both fruit flesh and foliage coloration, was used for genetic mapping because of its large size and the availability of a sufficient number of fruit. The red-fleshed parent, 91.136 B6-77, is an open pollinated seedling of the red-fleshed and red-leafed cultivar 'Redfield', itself derived from a cross between the cultivars 'Wolf River' and *Malus pumila *var. *niedzwetzkyana *(also with red flesh and foliage). For all 32 families, foliage colour was phenotyped once in early spring for orchard-grown trees for crosses made in 1998 – 2003 or in the glasshouse when plants were five weeks old for crosses made in 2004, when clear differences in foliage colour (red or green) could be observed. The fruit flesh colour phenotypes were assessed for the A194 population in each year from 2003 to 2005 and for the 'Geneva × Braeburn' family in 2006. Six fruit were selected from each tree, the flesh colour intensity was evaluated on a 0–9 scale (0 = white, 9 = intense dark red) on equatorial fruit section. If red colour was observed in the flesh in any of the six fruit, seedlings were designated either red cortex/red core or red cortex/white core depending on where in the fruit the red colour was found, or white cortex/white core. White cortex/red core was not found.

Two methods were employed for DNA isolation. For PCR-based marker development and testing, leaf tissue samples from a subset of 28 individuals from the A194 population that showed reproducible flesh colour were collected and extracted following the protocol described by Gardiner *et al. *[45]. For the screening of PCR-based markers over the whole population, leaf tissue samples were collected from all individuals and DNA extracted using an automated extraction protocol.

### Candidate gene selection

A set of candidate genes (Table 3) was selected according to three criteria: i/sequence homology to transcription factors that regulate the synthesis of anthocyanins or the anthocyanin biosynthetic pathway; ii/differential expression patterns between extreme phenotypes in both model systems and in apple [14,16]; iii/phenotype observed when the candidate genes were over-expressed in transformed model plant systems or mutated such as antirrhinum, petunia, maize and *Arabidopsis *[3,4,6,10]. A total of 13 candidate genes were used for marker development, with 12 selected from the HortResearch apple EST database and one isolated using degenerate primers (MdMYB10) as described in Espley *et al. *[16]. This set of 13 candidate genes was made of: four MYB TFs including genes homologous to Production of Anthocyanin Pigments 1 (PAP1), (MdMYB10; DQ267897), Transparent Testa 2/TT2 (MdMYB9; EB151663), a colour inhibitor (MdMYB17; CO867070) and a colour activator (MdMYB12; CV881251); two BHLH TFs including genes homologous to DELILA (MdBHLH33; DQ226451) and Transparent Testa 8/TT8 (MdBHLH3; EB128065); a gene homologous to ANL2 (MdANL2; EB176423) and a WD40 TF (MdTTG1; EB135547); five structural genes belonging to the anthocyanin biosynthetic pathway, including BANYULS (MdBAN, EG631316), Chalcone Synthase (MdCHS; EB140418), Dihydroflavonol 4-reductase (MdDFR; AF117268), Flavanone 3-hydroxylase (MdF3H; EB141429) and Leucoanthocyanidin dioxygenase (MdANS; EB127830).

### SNP and SSR detection

A hierarchal strategy was used to detect DNA polymorphisms in the candidate gene set. First, an automatic bioinformatics tool was used to search for single nucleotide polymorphisms (SNPs) and simple sequence repeats (SSRs) within the HortResearch apple EST database. The database was populated by ESTs generated by two large EST sequencing efforts, one carried out at HortResearch [17] and the other a NSF-funded project [46], as well as various smaller projects for which sequences have been deposited in GenBank. We identified the contigs corresponding to the selected candidate genes using BioView's query tools to mine for SNPs and SSRs in the EST alignments. Only biallelic SNPs that showed the same allele in at least two of the ESTs that made up the contig alignment were retained in order to avoid false SNPs that correspond to sequencing and cloning errors. For that reason, only the contigs with more than four ESTs were used for SNP mining. For the candidate genes that were not present in the EST database or that presented sequence alignments that were below our minimum EST threshold, a second approach was employed. PCR primers were designed to the candidate genes and used to amplify genomic DNA extracted from both parents of the mapping population. The PCR fragments obtained were then cloned, and six independent sequences were scrutinised for SNPs and SSRs using Vector NTI sequence alignment programme (Invitrogen, Carlsbad, CA, USA).

### Molecular marker development and PCR conditions

The identified SNPs were converted into dominant PCR-based molecular markers by designing allele-specific PCR primers [47] using Primer 3 software [48]. One of the PCR primers was positioned to have the SNP at the 3' end and an additional mismatch was inserted at the fourth base in from the 3' end. Two sets of primer pairs (Table 3) were developed to amplify each of the alleles specifically. For MdMYB10, one PCR primer was placed at the position of a 4 bp indel. A third primer was added to the MdMYB10 marker PCR mix to amplify a 1,182 bp PCR product used as a positive control for amplification (Figure 1). PCR reactions were carried out in 16.5 μl volume containing 1× PCR buffer mix (Invitrogen, Carlsbad, CA, USA), 1.3 mM MgCl_2_, 100 μM of each dNTP, 0.72% formamide, 10 μM of each primer, 0.5 U of Platinum^® ^*Taq *DNA polymerase (Invitrogen, Carlsbad, CA, USA) and 2 ng of genomic DNA. PCR amplifications were performed in a Hybaid PCR Express Thermal Cycler (Thermo Electron Corporation, Waltham, MA, USA) with conditions as follows: 94°C for 2 min 45 sec followed by 40 cycles at 94°C for 55 sec, 55°C for 55 sec and 72°C for 1 min 39 sec, and a final elongation at 72°C for 10 min. PCR products were separated on 0.9% agarose gels and stained with ethidium bromide.

For SSR markers, dinucleotide, trinucleotide and tetranucleotide repeats were identified and PCR primers designed to flank the repeated motifs using Primer 3 software. PCR product size was kept below 200 bp using PCR conditions described previously [36,37]. The amplicons were separated using a CePRO 9600 capillary electrophoresis system (Advance Analytical Technologies Inc., Ames, IO, USA).

### Candidate gene mapping

All markers (both SNPs and SSRs) were evaluated for co-segregation with red colour in the mapping population by screening both parents and six progeny representing the phenotypes extremes. When a putative co-segregation was identified, the marker was genotyped over all the progeny that had been phenotyped for fruit colour. If the association was confirmed, a partial genetic map was constructed around the candidate gene and the Mendelian trait, incorporating published SSRs [36,37]. In order to infer the genomic position of the remaining candidate genes (i.e. those not linked to red colour), the markers were screened in a population resulting from a cross between M.9 and 'Robusta 5' for which a genetic map is under construction (J-M. Celton and S.E. Gardiner, personal communication).

The mapping strategy used was the double pseudo-testcross [49]. Genetic linkage analysis was performed using Joinmap^® ^v3.0 [50] using the Kosambi mapping function with a LOD score of 6 for grouping.

## Authors' contributions

DC participated in SNP and SSR discovery and genotyping, mapping of the candidate genes, linkage analysis and manuscript preparation. CMC and CB participated in the mapping of candidate genes and LG 09 construction. ACA, RE and RPH selected the candidate genes. RNC developed the bioinformatics system used for SNP and SSR discovery. RKV and NCO developed the segregating populations and CJW and RKV collected the red flesh and foliage phenotype data. SEG was overall project leader. All authors read and approved the final manuscript.
